# Leveraging Google Earth Engine for Drought Assessment using Global Soil Moisture Data

**DOI:** 10.3390/rs10081265

**Published:** 2018-08-11

**Authors:** Nazmus Sazib, Iliana Mladenova, John Bolten

**Affiliations:** 1 Hydrological Sciences Branch, NASA Goddard Space Flight Center, Greenbelt, MD;; 2 Science Application International Corporation (SAIC), Lanham, MD;; 3 Earth System Science Interdisciplinary Center, University of Maryland, College Park, MD;

**Keywords:** Soil moisture, SMOS, SMAP, Google Earth Engine, Drought

## Abstract

Soil moisture is considered a key variable to assess crop and drought conditions. However, readily available soil moisture datasets developed for monitoring agricultural drought conditions are uncommon. The aim of this work is to examine two global soil moisture data sets and a set of soil moisture web-based processing tools developed to demonstrate the value of the soil moisture data for drought monitoring and crop forecasting using Google Earth Engine (GEE). The two global soil moisture data sets discussed in the paper are generated by integrating Soil Moisture Ocean Salinity (SMOS) and Soil Moisture Active Passive (SMAP) satellite-derived observations into the modified two-layer Palmer model using a 1-D Ensemble Kalman Filter (EnKF) data assimilation approach. The web-based tools are designed to explore soil moisture variability as a function of land cover change and to easily estimate drought characteristics such as drought duration and intensity using soil moisture anomalies, and to inter-compare them against alternative drought indicators. To demonstrate the utility of these tools for agricultural drought monitoring, the soil moisture products, vegetation- and precipitation-based products are assessed over drought prone regions in South Africa and Ethiopia. Overall, the 3-month scale Standardized Precipitation Index (SPI) and Normalized Vegetation Index (NDVI) showed higher agreement with the root zone soil moisture anomalies. Soil moisture anomalies exhibited lower drought duration but higher intensity compare to SPIs. Inclusion of the global soil moisture data into GEE data catalog and the development of the web-based tools described in the paper enable a vast diversity of users to quickly and easily assess the impact of drought and improve planning related to drought risk assessment and early warning. GEE also improves the accessibility and usability of the earth observation data and related tools by making them available to a wide range of researchers and the public. In particular, the cloud-based nature of GEE is useful for providing access to the soil moisture data and scripts to users in developing countries that lack adequate observational soil moisture data or the necessary computational resources required to develop them.

## 1 Introduction

The remote sensing advances made over the past three decades have radically improved our ability to obtain routine, global information about the amount of water present in the soil [1–3]. Several well-evaluated soil moisture (SM) data sets have proven useful for a wide range of applications, including weather and climate forecasting, monitoring of drought and wildfires, tracking floods and landslides, enhanced agricultural productivity [4–7]. Despite all the advantages the satellite-based soil moisture monitoring offers, such as global coverage, high accuracy, and high temporal frequency essential for operational applications, the shallow penetration depth of the microwave frequencies used for soil moisture monitoring remains a limiting factor [3]. In fact, many hydrological processes, forecasting and decision-making activities linked to soil moisture require knowledge of the root zone soil moisture information (RZSM). The latter is typically estimated using hydrologic land surface models, which are traditionally driven by some weather-related observations such as precipitation, temperature, etc. The credibility of the modeled RZSM estimates is strongly dependent on the quality of the forcing data [8,9].

Hydrologic data assimilation (DA) offers a way to reduce the impact of precipitation-related errors and enhance the quality of the modeled RZSM data through integrating satellite-based observations into the model. DA is essentially an optimal merging technique that results in an enhanced analysis product with improved accuracy over either of the parent products alone (i.e. the model data and the satellite observations). This paper focuses on the value of a combined model-satellite soil moisture data set for near-real time drought and crop condition monitoring developed in an effort to improve the RZSM information used by the U.S. Department of Agriculture-Foreign Agricultural Services (USDA-FAS).

One of the operational objectives of USDA-FAS is the development of global crop yield forecasts, which heavily employ modeled soil moisture to force crop yield models. These forecasts are generated utilizing information from the Crop Condition Data Retrieval and Evaluation (CADRE) Data Base Management System (DBMS), where soil moisture is an essential indicator used to assess crop health and monitor drought conditions, and evaluate their impact on expected end of season yields. The baseline USDA-FAS RZSM information is generated using the modified Palmer model (PM), which is a relatively simple two-layer water balance model driven by daily estimates of precipitation and temperature[10–12]. The PM model is highly susceptible to the quality of the precipitation forcing data, which are more error-prone over poorly instrumented areas [8]. Over a decade of research conducted in collaboration with USDA-FAS demonstrated that the agency’s model-based RZSM estimates produced by the PM can be improved by assimilating satellite derived observations [12,13]. Here we focus on the operational implementation of the DA enhanced PM using soil moisture retrievals from two passive microwave missions, the European Space Agency (ESA)’s Soil Moisture and Ocean Salinity (SMOS)[14] and the National Aeronautics Space Agency (NASA)’s Soil Moisture Active Passive (SMAP) [15]. SMOS and SMAP, launched in 2009 and 2015, respectively are the first two missions specifically designed to monitor near-surface soil moisture at a global scale using L-band frequency.

The goal of this paper is to announce the availability of these global soil moisture data sets and demonstrate their value for drought monitoring using Google Earth Engine (GEE). GEE is a web-based service that stores a petabyte archive of earth observations and related data and provides an efficient processing software which enables users to develop complex geospatial analyses and visualizations utilizing high-performance computing resources. The GEE capabilities have been utilized for a range of applications, including soil mapping, malaria risk assessment, and automated cropland mapping [16–19]. In this study, we demonstrate the value of the SMOS- and SMAP-datasets and web-based tools utilizing the global soil moisture data set generated using the satellite-enhanced PM available in the GEE data catalog. GEE and the available tools enable users to acquire, process, analyze and visualize earth observing data rapidly for any user specified region across the globe without downloading and processing a large volume of data on the user’s desktop. The web-based drought assessment tools alleviate the need for users to install and work with desktop data managing and processing software which are often labor intensive, time consuming and difficult to reproduce, thereby overcoming compatibility limitations and enhancing usability and reproducibility of the analyses and results.

The paper is organized as follows: Section 2 provides a detailed description of the soil moisture data, modeling approach and preparation steps for integrating the data into the GEE platform; Section 3 focuses on the functionality of the GEE tools developed for drought assessment using the satellite-enhanced PM global soil moisture data; Section 4 describes the application of the GEE tools over South Africa and Ethiopia; and Section 5 and 6 provide some discussion of the results and conclusion, respectively.

## 2. Data processing for GEE platform

**Figure 1:**
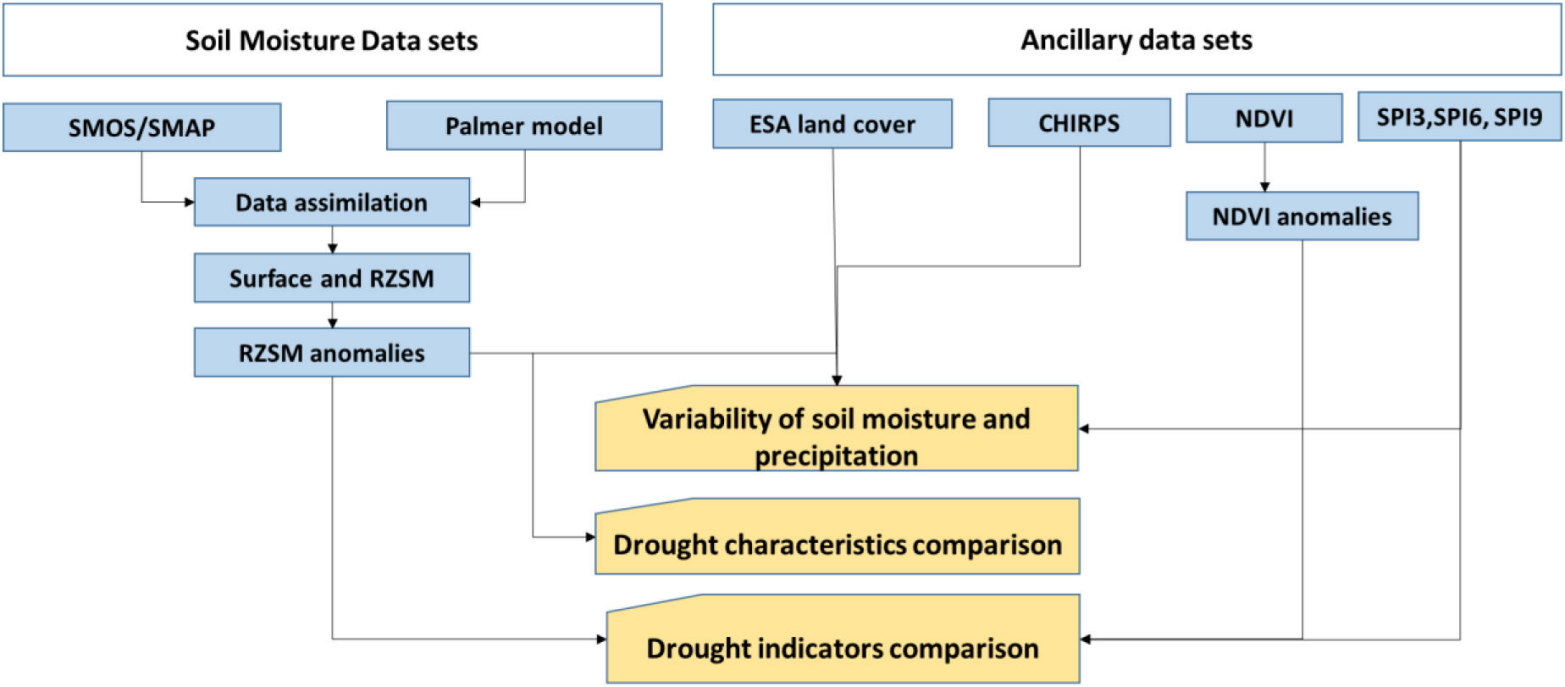
Schematic overview of methodological approach.

An overview of major methodological steps applied in this study is provided in Figure 1. First, we processed satellite-based soil moisture data sets to estimate surface and RZSM, and their anomalies. Then, we used RZSM and precipitation data to explore their spatial and temporal variability with different land cover types. Next, we estimated drought characteristics from RZSM anomalies and compare against other alternative drought indices. Details about these data sets are provided in Table 1. One of the primary goals of this study is to introduce global soil moisture data sets in the GEE, hence we provide details description of the soil moisture data sets in the following sub-section.

### 2.1. Soil Moisture data set

#### 2.1.1 The USDA-FAS Soil Moisture System: Palmer Model, Data Assimilation and Satellite Observations

##### a. Theoretical Basis

The two-layer Palmer Model used by USDA-FAS is a bookkeeping water balance model that accounts for the water gained by precipitation and lost by evapotranspiration [10]. The top layer is assumed to have 2.54 cm available water holding capacity at saturation, while the holding capacity of the lower layer varies depending on the depth of the bedrock. The model is driven by daily precipitation data and daily minimum and maximum temperature observations provided by the U.S. Air Force 557^th^ Weather Wing (formerly known as U.S. Air Force Weather Agency, AFWA). The AFWA data set is derived using multiple sources, inducting remotely sensed observations and gauge data acquired from the World Meteorological Organization (WMO). The model is enhanced by adding a data assimilation unit, which allows the routine integration of satellite-based observations into the model using a 1-D Ensemble Kalman Filter approach (EnKF) [11,12]. The purpose of this modification is to improve the PM RZSM information by integrating added value of the surface soil moisture retrievals to the model and examining their potential to correct for meteorological forcing uncertainty. A detailed description of Bayesian theory-based filtering, including the EnKF is beyond the scope of this paper, however, the methods are well-established and documented [26–30].

EnKF is a sequential Monte Carlo assimilation technique, where the model forecasts are optimally updated in response to the satellite observations via the Kalman gain (K). Operational implementation of EnKF requires some knowledge of the model uncertainty (Q) and the error of the satellite observations (R). Here, both of these parameters have been parameterized using some *a priori* knowledge. Given the above discussed and well-established dependence of the model accuracy on the uncertainty of the rainfall data, and the fact that the AFWA rainfall data set is rain-gauge corrected, R has been modeled as a function of proximity to WMO gauge station. Q on the other hand has been parameterized as a land cover type using published accuracy assessment analysis [31–37]. We discuss the implementation of the satellite enhanced PM using remotely sensed observations acquired from two L-band missions, SMOS and SMAP. Full technical description of these missions can be found in [14] and [38], respectively.

The corresponding SMOS and SMAP soil moisture estimates assimilated into the PM are derived using slightly different retrieval approaches, however, both systems and soil moisture products show similar performance and overall accuracy [39,40]. The global SMOS soil moisture data are operationally acquired from the NOAA Soil Moisture Products System (SMOPS), which are distributed at 0.25° grid spacing (https://data.nodc.noaa.gov/cgi-bin/iso?id=gov.noaa.ncdc:C00994; last accessed May 2018). SMAP offers a variety of soil moisture products (https://smap.jpl.nasa.gov/data/; last accessed May 2018). This study applies the L3 passive only SMAP soil moisture product. The data are routinely downloaded from the National Snow and Ice Data Center (https://nsidc.org/data/SPL3SMP/versions/4; last accessed May 2018). SMAP is distributed in EASE-grid 2 projection at 36 km grid spacing; therefore, the data have been pre-processed to match the model grid of 0.25°.

##### b. Operational Implementation

The satellite enhanced Palmer Model is set to run operationally on the NASA’s Global Inventory Modeling and Mapping Studies (GIMMS) Global Agricultural Monitoring (GLAM) system [23]. The model covers the Land Information System (LIS) domain (180°W − 180°E, 90°N-60°S) at 0.25° [41]. The system generates various soil moisture products, all are exported in GRIB format: surface and root zone soil moisture measured in [mm], profile soil moisture in [%] and surface and root zone soil moisture anomalies [–]. The latter represents standardized anomalies, which are calculated using following equation:

$$SMA\;=\;\frac{X_{SM}-\mu_{SM}}{\sigma_{SM}}.$$

Where, *X*_*SM*_ is the SMOS/SMAP soil moisture, *μ*_*SM*_ is the mean value, and *σ*_*SM*_ is the standard deviation of the SMOS/SMAP soil moisture. Each value shows the deviation of the current conditions relative to a long-term average standardized by the climatological standard deviation, where the climatology values are estimated based on the full data record of the satellite observation period over a 31-day moving window (e.g., climatology of a day of interest is calculated using the 15 days prior and 15 days after that day of year for the entire historical record). Negative anomaly values indicate that the current conditions are below average, while positive indicate surplus of water.

The system is executed daily as new AFWA and satellite observations become available. However, SMOS and SMAP provide complete global coverage every 3 days, therefore, the output generated from the satellite-enhanced PM is binned to 3-days composites. Once a new 3-days composite product is produced, the data are operationally pushed to USDA-FAS and the data are automatically displayed at the agency’s Crop Explorer web site. It should be noted that the SMOS-and SMAP-based systems are currently run independently and are expected to have slightly different climatologies given that each cover different time period (PM+SMOS: January 2010 to present; PM+SMAP: April 2015 to present).

### 2.2. Ancillary data sets:

Several additional data sets have been used in this study to explore the relationship between RZSM anomalies and meteorological drought indices as a function of land cover variability. The Climate Hazards Group Infrared Precipitation with Station (CHIRPS) data set, developed by the United States Geological Survey (USGS) in collaboration with Earth Resource Observation and Science (EROS) center is used to explore the spatial and temporal variability of the precipitation with different land cover types. CHIRPS is generated by integrating satellite imageries and in-situ gauge-collected observations. The daily rainfall data are distributed at 0.5° spatial resolution [42]. Vegetation type information was obtained from the ESA’s global land cover data developed by utilizing observations from Medium Resolution Imaging Spectrometer (MERIS) collected by the Environmental Satellite (ENVISAT)[43]. The land cover map includes 22 land cover classes as defined by the Food and Agriculture Organization of the United Nations (FAO) Land Cover Classification System (LCCS).

The Standardized Precipitation Index (SPI) is a meteorological drought index used to assess different drought characteristics. SPI represents the standardized deviation of the observed cumulative precipitation relative to the long-term precipitation average. In this study, SPI at 3, 6 and 9-month scales were obtained from the International Research Institute for Climate and Society (IRI) at Columbia University [22]. The SPI data set was derived from the monthly precipitation totals from the Climate Prediction Center’s (CPC) gauge –Outgoing Longwave Radiation (OLR) Blended global daily precipitation data. SPI was calculated by fitting a probability distribution to the long-term series of precipitation accumulation over the period of interest, where the resulting cumulative probability function is consequently transferred to a normal distribution. The monthly SPI data offers global coverage at spatial resolution of 1°.

The Normalized Difference Vegetation Index (NDVI) data were obtained from the Global Inventory Modeling and Mapping Studies (GIMMS) Global Agricultural Monitoring (GLAM) system. This dataset is derived using the Resolution Imaging Spectroradiometer (MODIS) Terra surface reflectance products, which are provided by National Aeronautics and Space Administration (NASA) Goddard Space Flight Center (GSFC) MODIS Adaptive Processing System (MODAPS) [44]. SPI and NDVI data sets are not available in the GEE public data catalog, hence, they were processed and ingested as personal assets in the GEE.

All data products used in this study have been averaged to monthly composites and then resampled to 1° grid spacing to ensure comparable temporal and spatial resolutions among the different datasets.

## 3. Google Earth Engine Tools:

We have developed several GEE tools which enable easy processing, analysis, and visualization of SMOS- and SMAP-based soil moisture data in the GEE platform. These tools can be arranged in three groups according to their functionality: (1) tools to process and ingest soil moisture data in the GEE data catalog, (2) tools to explore spatial and temporal variation of soil moisture and precipitation as a function of land cover, and (3) tools to estimate drought characteristics such as duration and intensity using soil moisture anomalies and inter-compare the latter against alternative drought indices. Detailed description of the individual tools is given below.

### 3.1. Data uploading routine:

**Figure 2:**
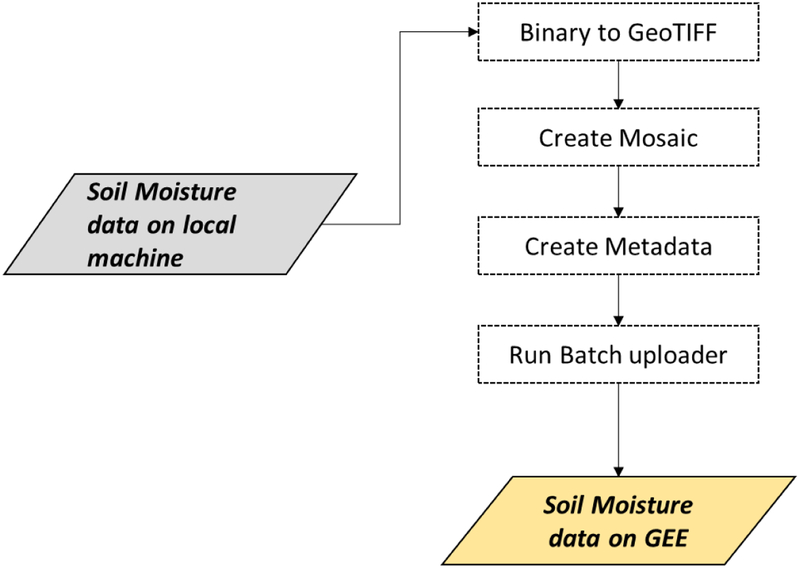
Ingestion of soil moisture data sets to Google Earth Engine. The gray and gold boxes represent inputs and outputs respectively.

The data upload routine has been designed to process, upload, and manage the SMOS- and SMAP-based soil moisture products in the GEE platform. This routine first converts the original soil moisture data stored in binary format into Georeferenced Tagged Image File Format (GeoTIFF) format as required by the GEE. Then, it creates a metadata file of the resulting imagery. The metadata is needed by the analysis routines, which is used to filter the data based on user specified spatial and temporal information. Next, the GEE Batch Asset Manager (https://github.com/tracek/gee_asset_manager) tool is used to upload bulk amount of data automatically in the GEE (Figure 2). An alternative uploading option is to use the Asset Manager option in the GEE. However, the latter is time inefficient for large data sets as it allows the user to upload a single imagery at a time.

### 3.2. Soil Moisture Exploration routine:

The soil moisture exploration routine has been specifically designed to assess the spatial and temporal variability of soil moisture from local to regional and global scale. This function first filters the soil moisture data based on user specified temporal and spatial criteria using GEE *‘filterDate’* and *‘filterBounds’*; functions.. Next, the subset data are grouped by month using ‘Filter.calenderRange’ function and aggregated from the original 3-days composites into monthly composites. Then, interactive monthly soil moisture plots are generated using the chart function by using *Chart image series* function in GEE, which can be viewed and exported in multiple formats, i.e. Comma Separated Values (CSV), Portable Network Graphics (PNG), etc. The multi-annual image collection can be further reduced to a long-term average image representing mean soil moisture for the region of interest, which can be visualized through GEE Google Maps. This routine also enables an assessment of the variability of soil moisture data as a function of land cover type. The ESA land cover data is clipped based on the user-defined region of interest and a histogram is plotted to estimate the major land cover types of the study region. Then, the monthly soil moisture values are filtered based on land cover class and interactive plots are generated for additional analysis and visualization.

### 3.3. Drought assessment routine:

**Figure 4:**
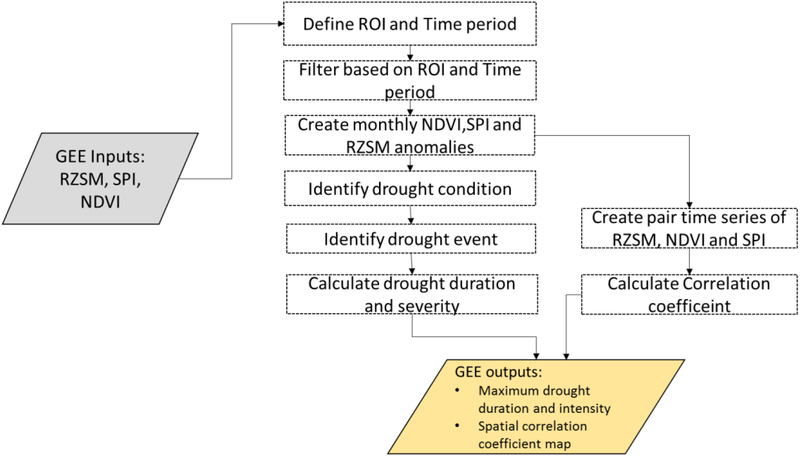
Data processing steps in the drought assessment routine using GEE. The gray and gold box represent GEE inputs and outputs respectively. The box identified by dotted line represents the process that run in the GEE server.

The drought assessment routine has been developed using GEE functionalities to compare various drought indicators based on specific drought characteristics such as percentage of month with drought conditions, maximum drought duration, drought severity, and intensity. In addition any drought indicators have been developed to monitor, predict and assess the severity of different drought types, which can be classified into two major categories of drought: meteorological and agricultural. Meteorological drought indicators are derived using precipitation data and have multiscale features that identify different types of drought condition. As root-zone soil moisture affects plants growth and productivity, RZSM anomalies are often used for quantifying and monitoring agricultural drought and capturing its impact on crop heath [4,45–47]. In this study, we used four drought indicators-SPI3, SPI6, SPI9 (meteorological drought indicators) and SMOS RZSM anomalies (agricultural drought indicator) to asses drought conditions. Here we focused on SMOS RZSM anomalies as it has longer observation period compared to SMAP, hence well suited to estimate drought characteristics and Pearson’s correlation coefficient. Positive values of RZSM anomalies, SPI3, SPI6 and SPI9 are masked out to identify only months with drought conditions, the corresponding number is divided by the total number of months to calculate the percentage of months with drought condition. Each product has been examined in terms of the following drought characteristics: drought duration (defined as the period during which the drought indices are continuously negative); drought severity (computed as the absolute value of the sum of all drought indices during a drought event); and drought intensity (calculated by dividing the severity by the drought duration) [48,49]. This routine also computes cross correlations between agricultural and meteorological based drought indices and allows to estimate the Pearson, Spearman and lag correlation coefficients using GEE *‘Reducer.pearsonsCorrleation’* function between the paired monthly time series of soil moisture anomalies and SPI as well soil moisture and NDVI (Figure 4).

**Figure 5:**
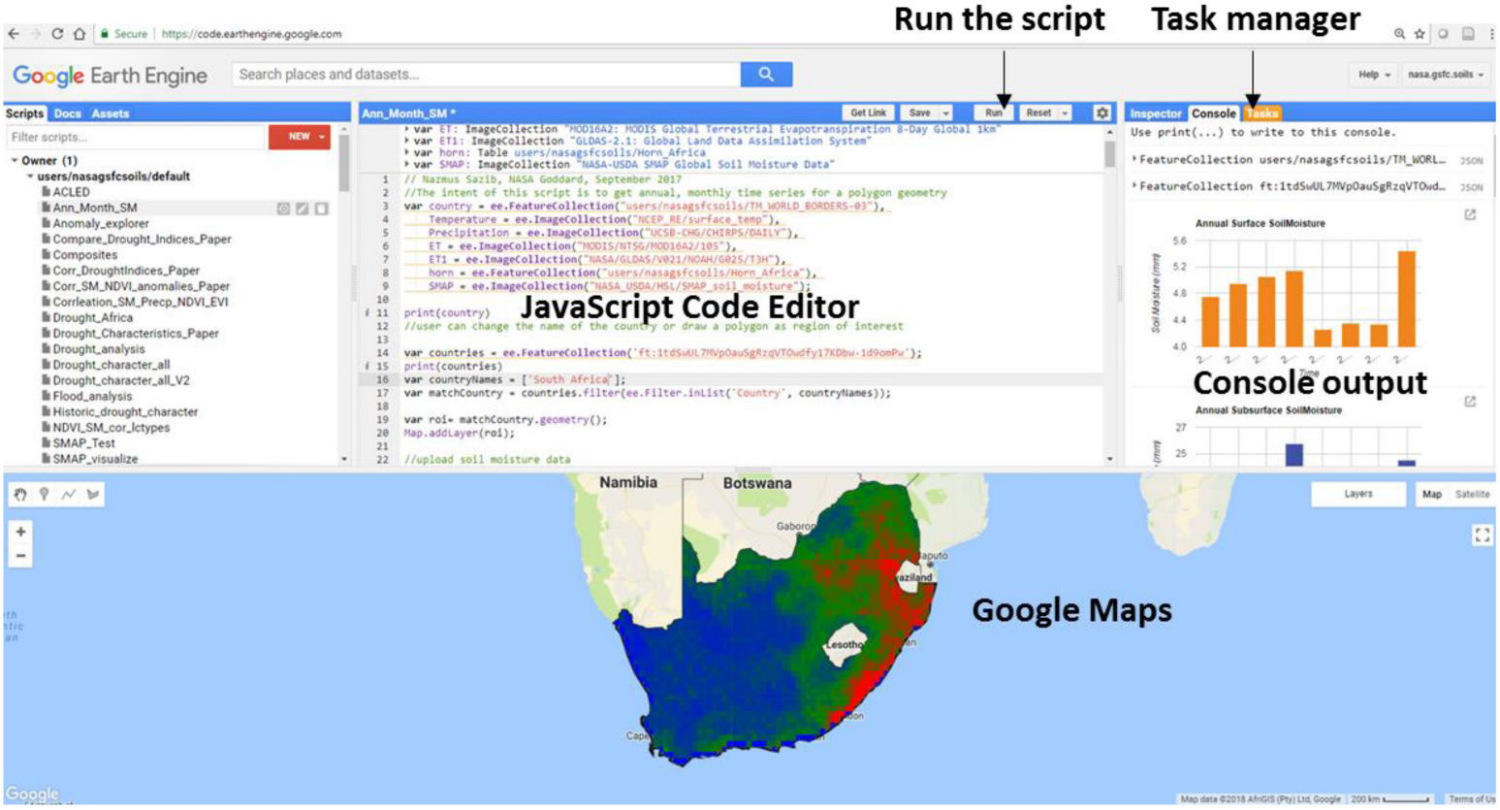
Components of Google Earth Engine code editor.

Developed tools are accessible through the links provided in the supporting materials, though potential users are required to register to access (https://code.earthengine.google.com/). Once the link is clicked, the user is presented with Earth Engine Code Editor which is a web-based Integrated Development Environment (IDE) for the Earth Engine JavaScript API (Figure 5). Then, user can execute the program by clicking the ‘run’ button located above the JavaScript code editor panel, if it does not start automatically. Once this has been done, time series plots and spatial map are displayed in the Console Tab and Google Maps respectively. The GEE outputs results can be exported by clicking on the run button in the Tasks tab located in the right panel next to the code editor.

## 4. Example Applications

The GEE tools described in the previous section have been implemented to evaluate the spatial and temporal dynamics of soil moisture and precipitation, and assess the ability of the drought indices described in the previous to capture severity, duration and intensity of drought events over South Africa and Ethiopia during 2010–2017.

Drought is common in South Africa and Ethiopia and occurs in all climate areas with varying degrees of intensity, spatial extent, and duration [50]. In recent years the spatial extent and frequency of drought have increased in this area causing significant water shortage, economic losses and adverse social consequences [51]. Therefore, better understanding of the climatology and drought characteristics over these areas is important in order to improve decision-making and aid activities aimed to mitigate the impact of drought. Our analysis is focused on the 2010–2017-time period, which was determined by the availability of the SMOS data sets. For this analysis, the soil moisture explorer routine is executed in the GEE code editor by clicking on the link provided in the supporting materials to generate spatial map and time series plots of the precipitation and soil moister over South Africa. Then, we run the drought assessment routine to estimate drought characteristics and correlation among different drought indices over South Africa. Next, we re-run both the soil moisture explorer and drought assessment routine for Ethiopia by changing the country name inside the script. The output results of the GEE are imported into ArcGIS [52] to add legend, scale and proper color scheme and R [53] to generate box plot of drought characteristics.

### 4.1. Spatial and Temporal variability of Precipitation and Soil Moisture:

**Figure 6:**
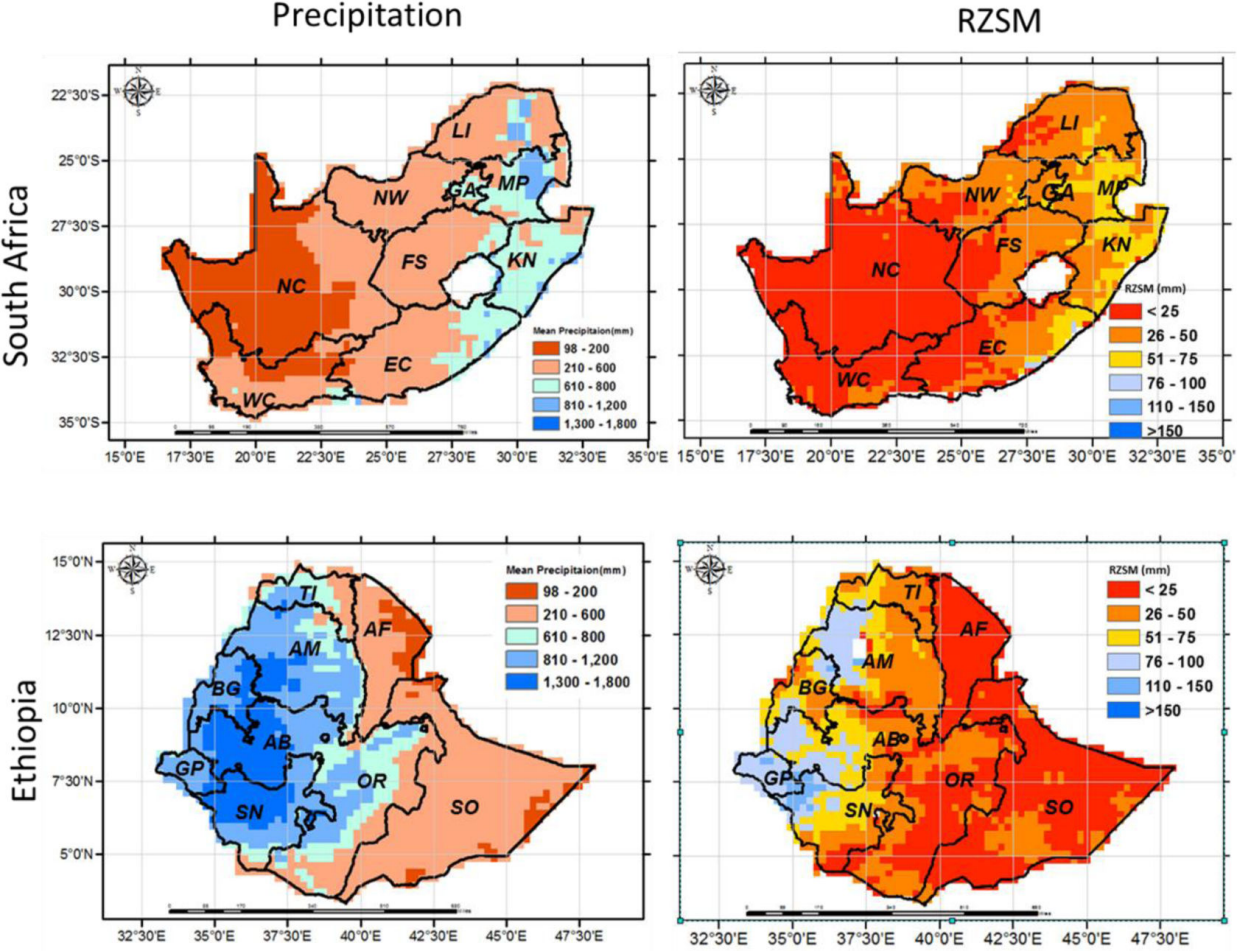
Spatial variability of precipitation (derived from CHIRPS precipitation data), and RZSM (derived from USDA-FAS soil moisture data) over South Africa (top row) and Ethiopia (bottom row) for the period of 2010–2017. The locations of the each province of South Africa (NC: Northern Cape, WC: Western Cape, EC: Eastern Cape, NW: North West, FS: Free State, KN: KwaZulu-Natal, MP: Mpumalanga, GA: Gauteng, LI: Limpopo) and Ethiopia (SO: Somali, OR: Oromia, SN: Southern Nations, GP: Gambela Peoples, BG: Benshangul-Gumaz, AM: Amhara, TI: Tigray, AF: Afar, AB: Addis Ababa) are also indicated.

We first examined the long-term spatial distribution of the precipitation and RZSM and then analyzed the variability of those variables with different land cover types. Spatial variability of rainfall and RZSM over South Africa and Ethiopia are shown in Figure 6. Both variables exhibit high regional variability. In South Africa, generally the mean annual precipitation increases from west to east with the maximum rainfall (680 mm) occurring over the Mpumalanga and KwaZulu-Natal, while minimum rainfall (172 mm) falls over the western part of the country. The spatial variability captured by the RZSM reflects the precipitation variability showing wetter SM conditions in the east and dryer in the west (Figure 1, top row). The topographical variability significantly influences the spatial distribution of the precipitation and the soil moisture in Ethiopia. For example, the rainfall and soil moisture values are higher over the highland areas located in the central and north-western portion of the country, while the lowland areas located in the eastern part of the country are associated with lower rainfall amounts (Figure 1, bottom row).

**Figure 8:**
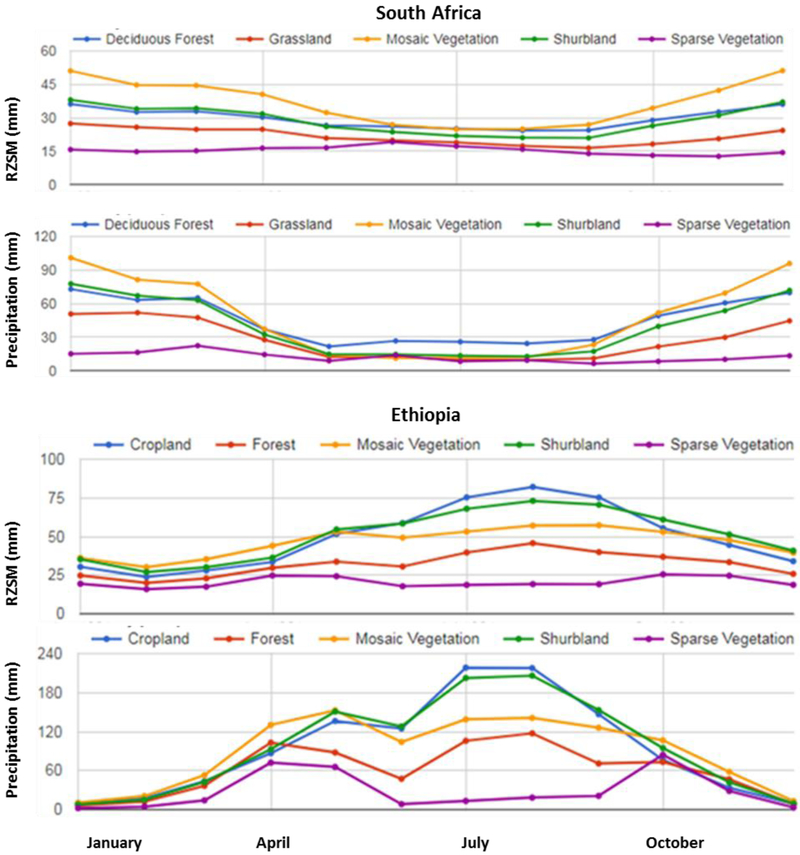
Monthly variation of soil moisture and rainfall for different land cover types over South Africa (top) and Ethiopia (bottom).

The monthly precipitation over Ethiopia and South Africa is driven mainly by the position of Intertropical Convergence Zone (ITCZ), which changes over the course of year [54,55]. A majority of the rainfall in Ethiopia falls during the summer seasons when the ITCZ is at its most northern position, however, the amount of rainfall also varies as a function of land cover. For example, forest, cropland, grassland and shrub land show identical rainfall patterns with one main wet season (June to September), and a secondary wet season (February to May), where the highest rainfall occurs during the month of September (Figure 8). Over sparse vegetation, the major rainfall falls during the summer and winter season as most of this land cover is located in the southern part of the country, where the rainfall timing is associated with ITCZ, which passes through the southern position of the equator at that time. The monthly RZSM follows the rainfall distribution reaching the wettest soil moisture conditions during the month of September. The position of ITCZ also results in two distinct seasons in South Africa - a wet and dry season roughly from November to April, and May to October, respectively. The monthly soil moisture time series captures this seasonality, as seen in Figure 2. The monthly rainfall and soil moisture time series across South Africa vary with land cover, where regions covered by the mosaic and sparse vegetation receive highest and lowest amount of precipitation and soil moisture, respectively (Figure 8).

### 4.2. Comparison of Drought characteristics

**Figure 9:**
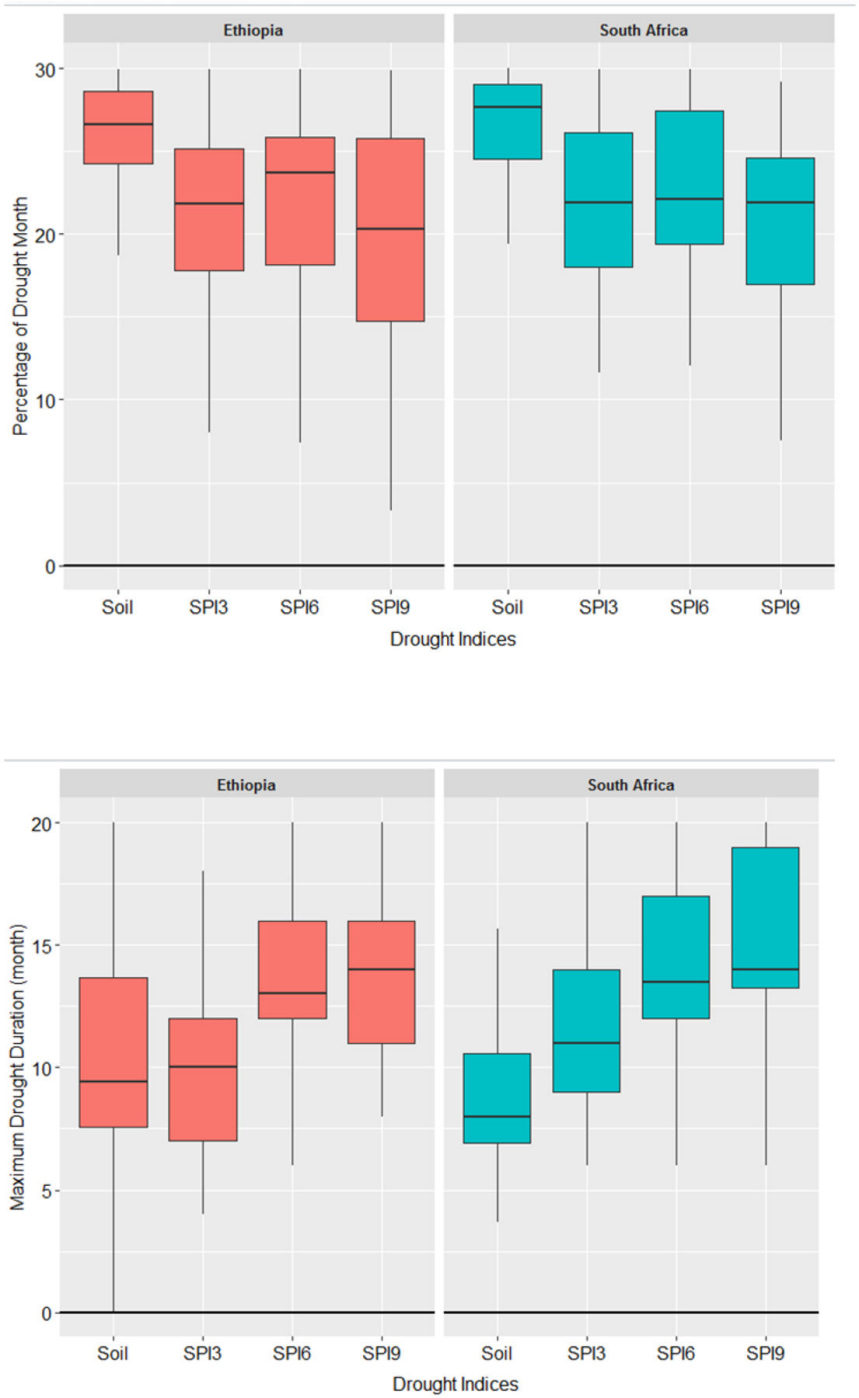

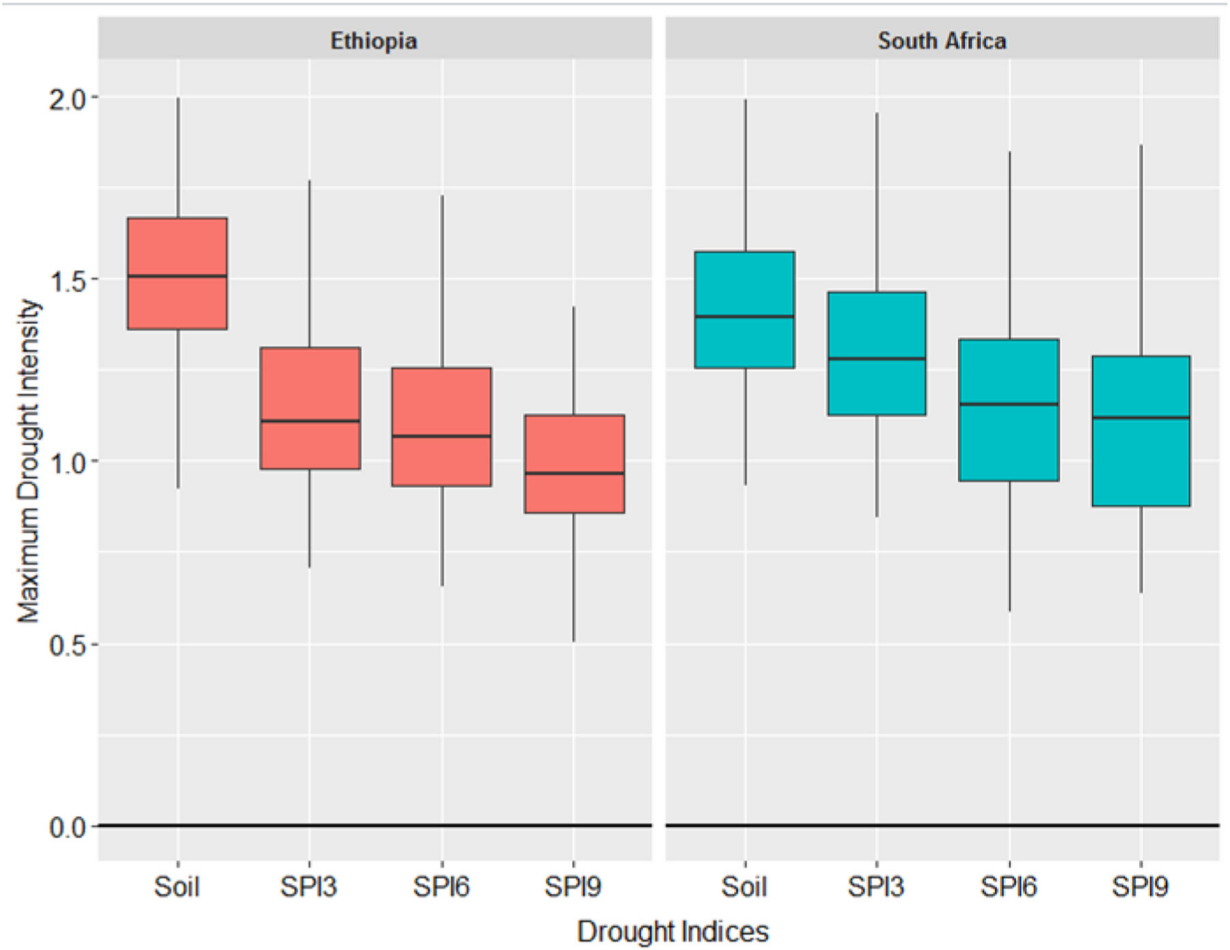
Comparison of percentage of months with drought condition, maximum drought duration and drought intensity over Ethiopia and South Africa for multiple drought indices. The center line of each boxplot depicts the median value (50th percentile) and the box encompasses the 25th and 75th percentiles of the sample data. The whiskers extend from q1 − 1.5 × (q3 − q1) to q3 + 1.5 × (q3 − q1), where q1 and q3 are the 25th and 75th percentiles of the sample data, respectively

The RZSM anomalies indicated higher percentage of months with drought conditions compared to SPI (SPI3, SPI6 and SPI9) over both study regions (Figure 9). Over South Africa, the average percentage of drought events identified in the RZSM anomaly data was 27%, which is 6% higher than the drought events captured by the SPI3. Additionally, among the rainfall-based drought indices, the SPI9 had the lowest percentage of months with drought events compared to the SPI3 and SPI6. This is in line with other studies [48], where the author found that agricultural-based drought indicators depict relatively larger values of drought months compared to the meteorological drought indices. The maximum drought duration varied among the different drought indicators. Based on our analysis, the maximum drought duration appeared to be higher in the meteorological-based indices than the agricultural-based indices. This is primarily because the meteorological-based drought indices integrate the drought condition over longer period of time than the agricultural-based drought indices [48]. The drought intensity was found to be higher in the agricultural-based drought variables and lower in the metrological-based drought indices. This example demonstrates the capability of the drought assessment tools, which can help to better assess the drought conditions.

### 4.3. Correlation between soil moisture and NDVI anomalies

**Figure 10:**
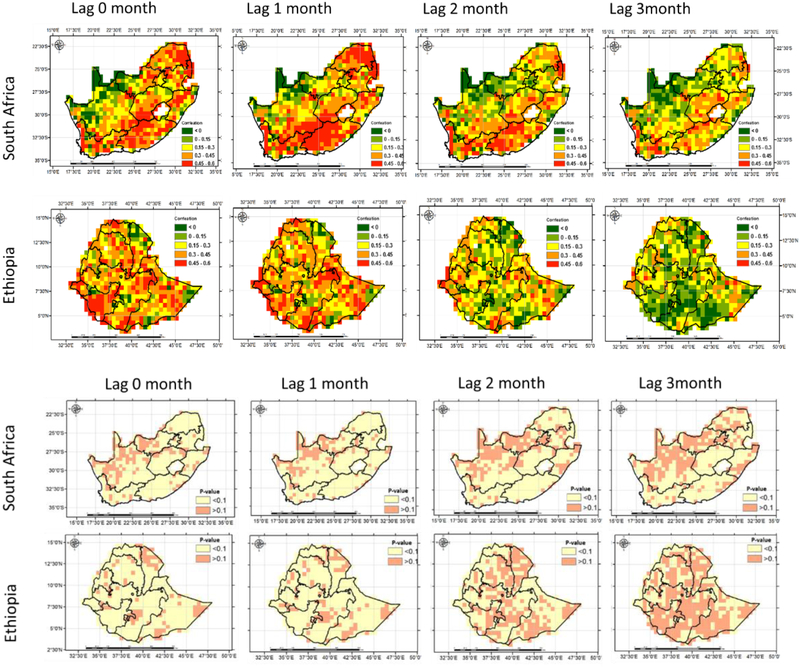
Correlation coefficient (top) and p-value (bottom) between RZSM and NDVI anomalies for different lag times.

Variations in RZSM substantially influence the vegetation dynamics (i.e., NDVI), which is a widely used vegetation index. Therefore, correlation analysis between RZSM and NDVI anomalies is important to understand the impact of changes in soil moisture on vegetation growth, which can be effectively utilized for early warning of time and areas of increased food insecurity [42,56]. The correlation of the RZSM and NDVI anomalies varied with the geographic location and the degree of lag time. The highest positive correlation coefficients and confidence level (i.e. p-vale < 0.1) are observed when soil moisture change is concurrent or precedes the change in NDVI by one month. In most of the locations NDVI and RZSM anomalies have positive correlations, however some regions indicate negative correlation at higher lags due to coincidence of negative NDVI anomalies with positive soil moisture anomalies [57]. In South Africa, the semi-arid Western Cape and Eastern Cape show higher coefficients compared to other parts of the country as the vegetation growth in those regions has high reliance on root zone soil moisture. [58,59]. No spatial variability in the lag correlation values was observed over Ethiopia (Figure 10). We further investigated the variation of the soil moisture and NDVI relationship as a function of major land cover types. The highest agreement was found over areas covered by grass land for both study areas, while the lowest agreement was achieved over the shrub covered areas in Ethiopia and South Africa (Table 2). This is partly due to the fact that grassland roots are located on the shallow depths and are more sensitive to changes in soil moisture than deep rooted plants such as shrub.

### 4.4. Correlation among soil moisture anomalies and meteorological based indices

**Figure 11:**
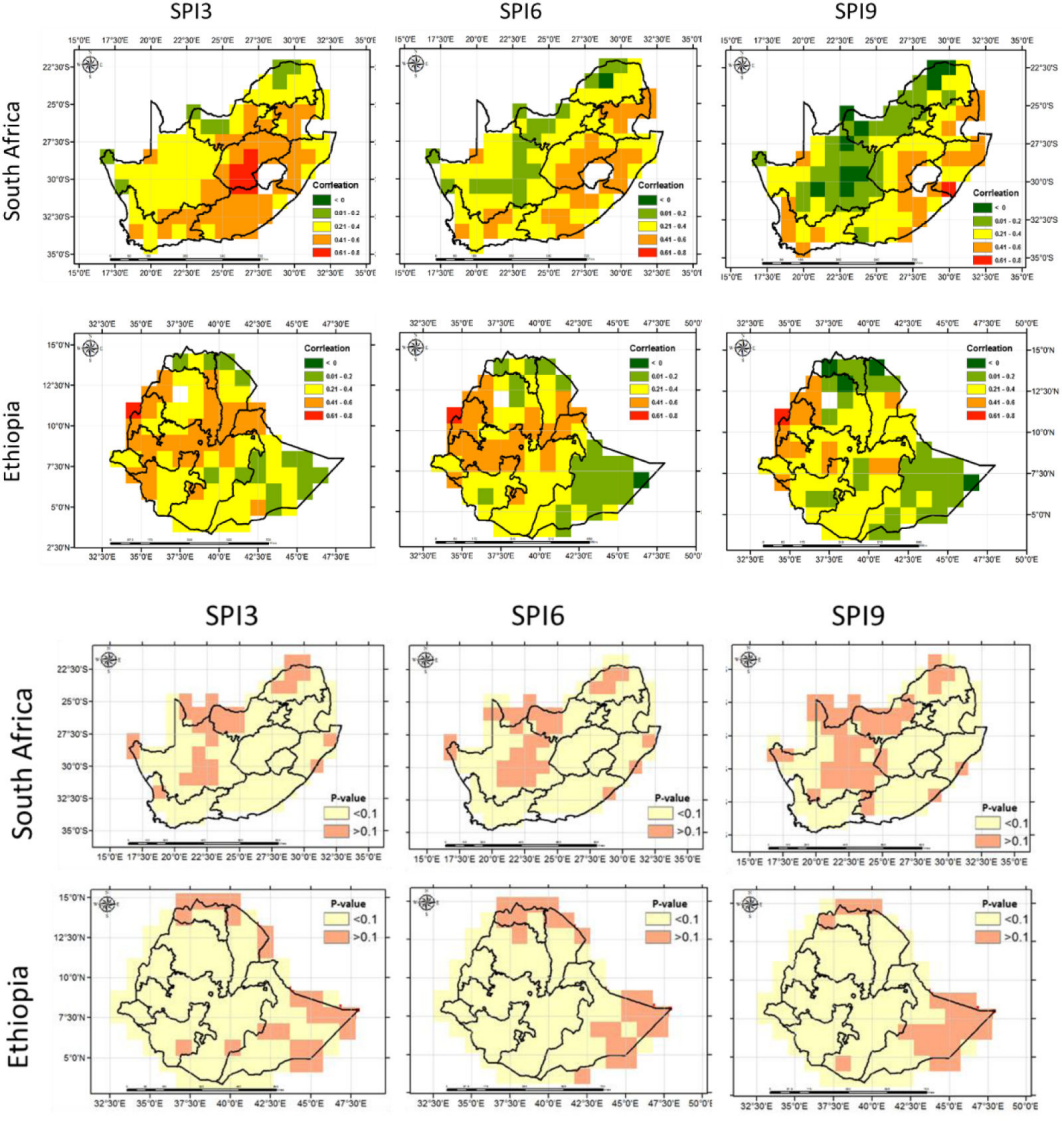
Spatial variation of Pearson correlation coefficients (top) and p-value (bottom) of RZSM anomalies with meteorological based drought indices.

A correlation analysis was carried out between RZSM anomalies and meteorological based drought indicators to evaluate how well the meteorological based drought indicators represent agricultural-based drought. Such information could be used to help indicate times and areas that are likely to experience agricultural stress. It is envisaged that such approaches will improve drought monitoring and early warning systems that rely mostly on meteorological indicators [60]. The GEE-based inter-comparative analysis between soil moisture anomalies against SPI showed high agreement and alludes to the value of combining such datasets to compliment a regional drought assessment that incorporates both meteorological and agricultural drought. Over both study regions SPI3 had higher correlation values compared to SPI6 and SPI9 (Figure 11), which indicates that SPI3 captures more of the agricultural drought. The performance of the meteorological drought indices varied spatially. In case of South Africa, the correlation values were relatively higher and statistically significant (p-value < 0.1) in the Western Cape and Eastern Cape compared to the Northern Cape of the region. The spatial distribution of the correlation for all meteorological droughts in Ethiopia have similar pattern, where higher and lower correlation values are generally distributed over the north – west and north-east side of the country respectively. The highest correlation between the soil moisture anomalies and meteorological drought indicator are associated with the cropland, which is consistent with [61], who showed that a 3-month SPI has the highest correlation with vegetation growth on croplands of the mid latitude U.S. Great Plains.

## 5. Discussion

Highest correlation of SPI3 with the RZSM anomalies indicated that short time meteorological drought represents the agricultural drought better compare to long-term meteorological-based indicators such as SPI6 and SPI9. The impact of meteorological drought on vegetation is cumulative meaning that vegetation does not respond instantaneously to the precipitation changes. The three-month SPI, which captures the precipitation pattern not only for the specific month of the interest, but previous two months as well results in highest correlation between SPI and soil moisture anomalies. On the contrary, the 12 and 6-month SPI values reflect precipitation patterns for annual and the entire growing season respectively and tend to diminish the variance in the precipitation data and smooth the SPI values results in lower correlation values [61]. The relationship between soil moisture and rainfall anomalies were also explored by Sims et al.[62] in the North Carolina where the author suggests that SPI on a scale of 2–3 months yielded highest correlation with soil moisture anomalies.

Our results indicate lower and higher correlation between RZSM anomalies and SPI based indicators in the dry and wet regions respectively, which could be related to the rainfall amount and soil types. The dominant soil types in the wet regions are clay and clay loam, which have higher water holding capacities, which could result in slower response to the rainfall, therefore soil moisture on a specific month would be more dependent on the previous month’s rainfall. On the other hand, the arid region shows quicker response of the rainfall anomalies due to dry soil conditions and limited water holding capacity of the sandy soils that covers that region. Therefore, soil moisture in a specific month has a smaller dependence on previous month compare to the wet region [63].

In general, the land cover type has a significant impact on the relationship between RZSM anomalies and other drought indicators. For example, shrub land exhibits lower correlation values compared to the cropland which could be due to the fact that cropland roots are located in the shallow depths and are more sensitive to changes in soil moisture than deep-rooted plants such as shrub. Similar observations were made by Camberlin et al. [64] and Huber et al. [65] for Africa, by Li et al.[61] for China and by Wang et al.[66] for the US-American central Great Plains. We also notice a delayed response of NDVI to RZSM anomalies for the shrub land over South Africa which might be related to soil texture and soil moisture amount as most of the shrub land are located in the wet region of the country characterized by more clay soils leading to slower response [67]. This is consistent with the findings of Wang et al. [66] who showed that the NDVI at humid sites takes longer time to response compare to the arid sites.

## 6. Conclusions

Soil moisture data are recognized as a fundamental physical variable that can be used to address science and resource-management questions requiring near real-time monitoring of the land-atmosphere boundary, including flood and drought monitoring, and regional crop yield assessment. This study introduced new sets of near real-time global soil moisture data and demonstrates the potential of GEE web-based tools and soil moisture data to assess regional drought condition. In general, meteorological drought indicator, SPI3, gives higher correlation values compared to SPI6 and SPI 9 with the RZSM anomalies. When, comparing the drought characteristics, RZSM anomalies exhibit relatively larger drought duration but smaller drought intensity compared to the meteorological-based drought indicators. The NDVI- RZSM anomalies are influenced by the vegetation cover, specifically shallow rooted plants that are more sensitive to soil moisture changes compared to deep rooted plants. The methods demonstrated here can be applied to other areas requiring early warning of food shortage or improved agricultural monitoring to help provide greater economic security within the agriculture sector.

Incorporating the global soil moisture data into GEE data catalog enables users to efficiently and quickly acquire and process large amount of data. The available tools allow easy analysis, visualization, and interpretation of the data. To this end, these GEE-based tools could enable scientists, policy makers and the general public to explore spatial and temporal variation of soil moisture information and drought conditions for any location in the world with minimal data processing or data management. In addition, all tools are easily transferable and can be used to explore spatial and temporal dynamics of other climate variables such as temperature and evapotranspiration. GEE does not require any additional software installation, which helps to overcome compatibility limitations and allows user to access the available codes and data from any computer connected to the internet. This significantly increases the data and tools usability and applicability. The GEE tools and the soil moisture data are open source and are freely available, which can enable users to use, modify, and suggest future improvements for both the tools and the data.

Although the GEE offers many benefits, it has limitations too. First, it requires basic knowledge of Python and Java script and users with limited programming knowledge might have a steep learning curve. Second, users are sometimes required to export the analyzed results to perform additional analyses due to limited functionalities and plotting options in the GEE. Finally, debugging the code is challenging as the user created algorithms run in the Google cloud distributed over many computers. Despite these limitations, the data distribution and processing approach offered by the GEE platform can be very beneficial, specifically for the developing countries that are typically data poor areas and lack high performance data processing platforms for drought monitoring or crop forecasting.

## Figures and Tables

**Figure 3:**
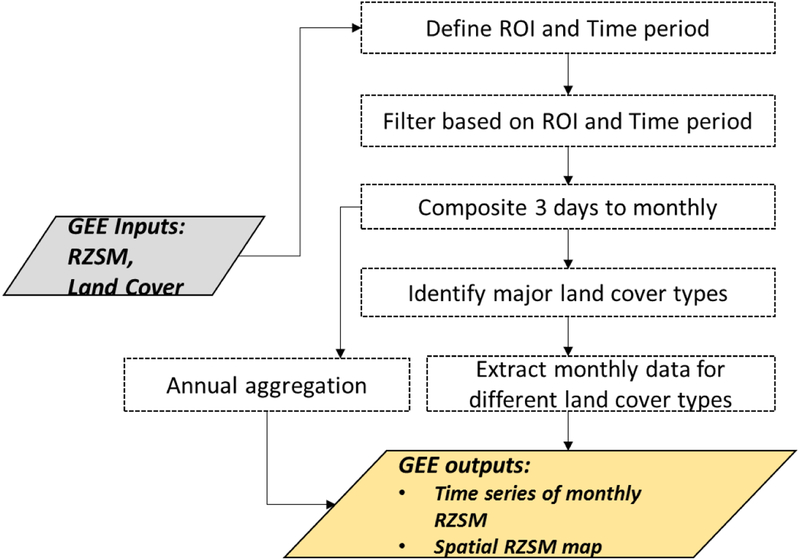
Data processing steps in the soil moisture exploration routine. The gray and gold boxes represent GEE inputs and outputs respectively. The box identified by dotted line represents the process that run in the GEE server.

**Figure 7:**
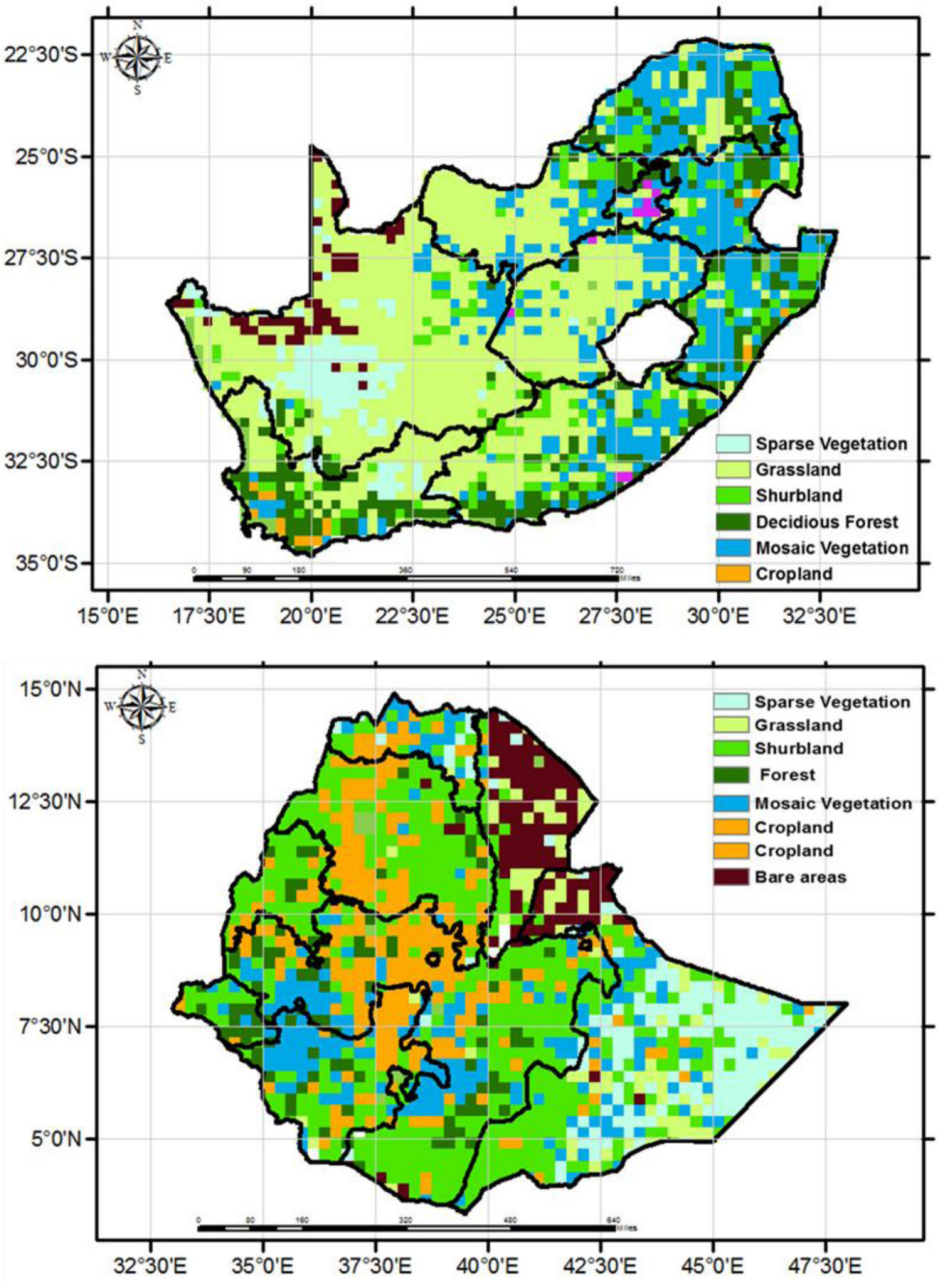
Land cover of South Africa (top) and Ethiopia (bottom).

**Table 1:** Datasets used in this study

No	Name	Spatial Resolution	Temporal Resolution	URL
1	NASA-USDA Global Soil Moisture Data	0.25°	3 day	[20]
2	NASA-USDA SMAP Global Soil Moisture Data	0.25°	3 day	[21]
3	SPI	1°	monthly	[22]
4	NDVI	0.00225°	8 days	[23]
5	CHIRPS Pentad: Climate Hazards Group InfraRed Precipitation with Station Data	0.05°	5 days	[24]
6	Global Land Cover Map	0.00275°		[25]

**Table 2:** The Pearson’s correlation coefficient computed between agricultural-based drought indices and meteorological-based drought indices for different land cover types.

	Ethiopia				South Africa			
Land cover	*Lag0*	*Lag1*	*Lag2*	*Lag3*	*Lag0*	*Lag 1*	*Lag2*	Lag 3
Crop Land	0.32	0.31	0.20	0.13	0.31	0.32	0.25	0.25
Grass Land	0.34	0.32	0.21	0.11	0.37	0.38	0.28	0.23
Shrub Land	0.32	0.30	0.20	0.11	0.24	0.29	0.23	0.18

**Table 3:** The Pearson’s correlation coefficient computed between agricultural based drought indices and meteorological based drought indices for different land cover types.

	Ethiopia			South Africa		
Land cover	*SPI3*	*SPI6*	*SPI9*	*SPI3*	*SPI6*	*SPI9*
Crop Land	0.41	0.42	0.32	0.43	0.39	0.34
Grass Land	0.37	0.36	0.29	0.37	0.30	0.23
Shrub Land	0.25	0.21	0.16	0.37	0.28	0.20
